# Down syndrome versus dementia

**DOI:** 10.1002/alz.70204

**Published:** 2025-06-19

**Authors:** Eden Rapp

**Affiliations:** ^1^ School of Public Health Boston University Boston USA

## 1 MEMORY LOSS

Hi, my name is Eden Rapp. I am 28 years old, and I just graduated from a 3 year post‐secondary program called Shepherds College in June 2024 in Wisconsin, where I studied Culinary Arts.

I have been a part of the Boston University Co‐Research team for about two and a half years. We research different health problems for people with Down syndrome so that we can strive to be more independent. We just published a study about mental health.

If people with Down syndrome live long enough, they might get dementia. So far in my life, my experience with dementia comes from my grandmother. I learned some important things about dementia.

My grandmother had both not so good short‐ and long‐term memory loss. Sometimes she could recall some things, but only in bits and pieces. Like she forgot who I was, and according to her, I was her servant that she had back in China. When my family was around, she would be nice to me, but when my family was doing other things, she treated me differently.

## 2 BEING TERRITORIAL

When you have dementia, it can be controlling at times and can change a person. My grandmother hardly let anyone come near her; only four people made her feel safe and secure: my mom, my dad, my uncle, and her dog. It made me feel invisible.

## 3 BEING DISORIENTED

Sometimes, having dementia is like playing telephone, where you say something one way, but then something completely different comes out of your mouth, and then it goes away like lightning. So, my grandmother had that problem, so we found that writing on a sticky note was very helpful, so if we didn't want to repeat ourselves and get annoyed, we would refer to the sticky note. That wouldn't work sometimes, and I would need to change the subject and calm her down by playing two musicals that she loved, The Sound of Music and My Fair Lady.

Sometimes she would leave the house and wander around until she made it back home successfully. For example, when we were at my great aunt's house in Connecticut, my grandmother forgot where she was, and she did not feel safe. She knew she wasn't around her safety net, so she got up and wandered away. By the time I woke up, my mom and my great uncle found her in the hospital after she was picked up by the police. So, sometimes being disoriented can be dangerous for someone with dementia. So, finding and using appropriate safety nets is really important to help someone who can easily be disoriented.

Even though she had dementia, there were still ways she was true to herself. Her love for music played a big part in her life, and at the end of her life, it kept her connected to me. The only time that she would say my real name and not call me her servant was when I was practicing piano or when my brother would practice singing or trumpet.

For me personally, it was hard to love and forgive my grandmother, but then I learned something that helped me change my mind. My mom taught me that “When you have dementia, you are not your true self, the true self is just buried like a gem that you find in a rock.”

It took me a long time to realize she was right. The best way to support is just being there for that person.

So far in my life, my experience with dementia is from my grandmother, but I know that having Down syndrome means I might be more likely to have dementia. My focus right now is on getting a job and not so much dementia, but it can happen even if it will be in 30 years.

There are still important things that young adults with Down syndrome can do to prevent and delay dementia.

1. Not being isolated and having a support network
2. Not feeling afraid to do things on your own
3. Taking care of yourself physically, emotionally, and spiritually
4. Eating healthy
5. Having an active mind

Because of my experience with living with my grandmother and knowing that people with Down syndrome can get dementia, I am more motivated to exercise my brain with my word search books and reading, which can decrease the risk of getting dementia.

As someone with Down syndrome, I think that it is important to share our experiences to make this world a better place. For example, I had the chance to lobby for two bills: the Transformation to Competitive Integrated Employment Act and the Marriage Equality for Disabled Adults Act, which will help people with Down syndrome. It is important for people with Down syndrome to have a voice and to speak up, including as researchers, because in this world, most people view us as a label, like we can't do much or that we will never be an asset to society.

So, the bottom line is that people with Down syndrome get dementia, but also have other issues they are worried about when they are younger. Someone wise once told me that dementia is like an ocean wave. It comes and it goes out like a colander with no filter. We don't have a cure yet, but for young adults with Down syndrome, I say choose to learn to take on individual challenges and choose to try new tasks and skills, one step at a time.


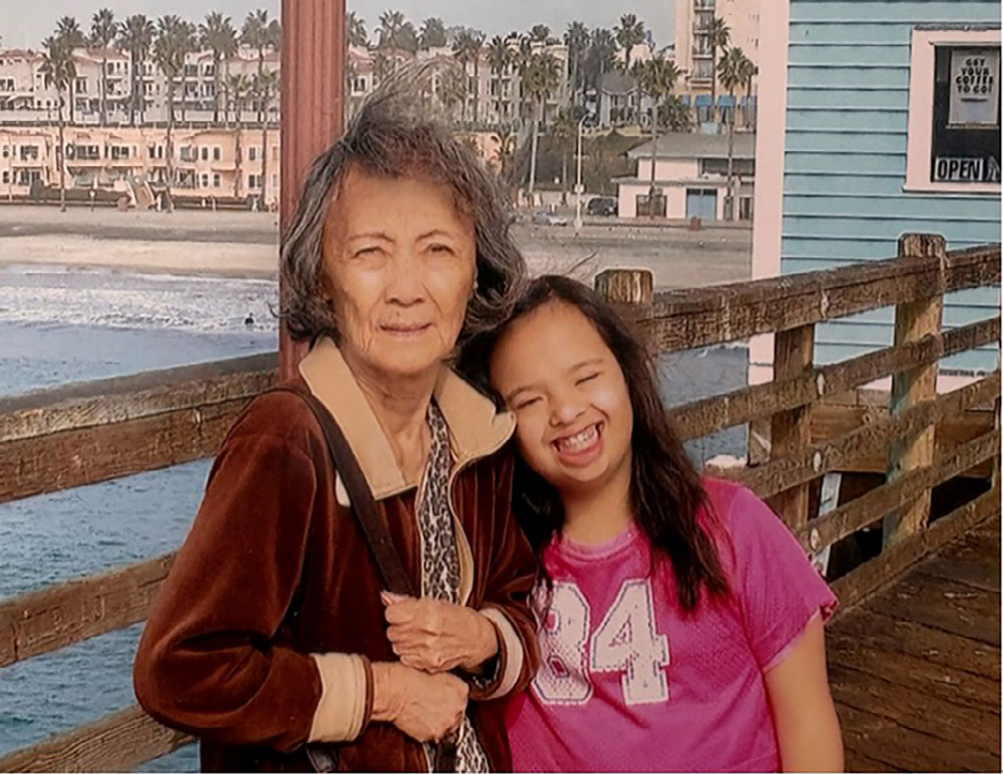
.

Picture 1 Eden Rapp and her grandmother, Robin Chan

## CONFLICT OF INTEREST STATEMENT

The author declares no conflicts of interest; conflicts of interest author disclosures are available in the Supporting Information.

## Supporting information



Supporting Information

